# Beyond carbon and nitrogen: guidelines for estimating three‐dimensional isotopic niche space

**DOI:** 10.1002/ece3.2013

**Published:** 2016-03-09

**Authors:** Sam Rossman, Peggy H. Ostrom, Forrest Gordon, Elise F. Zipkin

**Affiliations:** ^1^ Department of Integrative BiologyMichigan State UniversityEast LansingMichigan48824; ^2^ Ecology Evolutionary Biology and Behavior ProgramMichigan State UniversityEast LansingMichigan48824; ^3^ Hubbs‐Sea World Research Institute3830 S. Highway A1A #4‐181Melbourne BeachFlorida32951; ^4^ Department of MathematicsLouisiana State UniversityBaton RougeLouisiana70803

**Keywords:** Bayesian analysis, community ecology, niche, stable isotopes

## Abstract

Isotopic niche has typically been characterized through carbon and nitrogen ratios and most modeling approaches are limited to two dimensions. Yet, other stable isotopes can provide additional power to resolve questions associated with foraging, migration, dispersal and variations in resource use. The ellipse niche model was recently generalized to n‐dimensions. We present an analogous methodology which incorporates variation across three stable dimensions to estimate the significant features of a population's isotopic niche space including: 1) niche volume (referred to as standard ellipsoid volume, SEV), 2) relative centroid location (CL), 3) shape and 4) area of overlap between multiple ellipsoids and 5) distance between two CLs. We conducted a simulation study showing the accuracy and precision of three dimensional niche models across a range of values. Importantly, the model correctly identifies differences in SEV and CL among populations, even with small sample sizes and in cases where the absolute values cannot precisely be recovered. We use these results to provide guidelines for sample size in conducting multivariate isotopic niche modeling. We demonstrate the utility of our approach with a case study of three bottlenose dolphin populations which appear to possess largely overlapping niches when analyzed with only carbon and nitrogen isotopes. Upon inclusion of sulfur, we see that the three dolphin ecotypes are in fact segregated on the basis of salinity and find the stable isotope niche of inshore bottlenose dolphins significantly larger than coastal and offshore populations.

## Introduction

An ecological niche is an *n*‐dimensional hypervolume encompassing the entirety of an organism's interactions within its biotic and abiotic environment (Hutchinson 1978). The ecological niche concept has played a pivotal role in advancing our understanding of how species utilize and compete for resources, invade new environments, and evolve through time (McGill et al. 2006). Yet estimating the totality of a species' niche is difficult given real‐world constraints. For this reason, niche space is frequently divided into scenopoetic axes, which include environmental resources (e.g., habitat use), and bionomic axes, which relate to trophic interactions and ecological function (e.g., predation) (Hutchinson 1978). Stable isotope approaches to examining food webs provide a fast and efficient method of studying both scenopoetic and bionomic aspects of niche space. Variation in stable isotope values equates to variation in resource use and thus provides a means to estimate some components of niche space, referred to as the isotopic niche (Newsome et al. 2007). Isotopic studies generally focus on comparing and contrasting carbon and nitrogen stable isotope ratios (Newsome et al. 2010; Layman et al. 2012). Carbon isotope ratios (^13^C/^12^C) are controlled by primary production at the base of the food web and are thus predominately related to scenopoetic axes. For example, due to differences in photosynthetic pathways, C4 plants generally have higher carbon isotope values (*δ* ^13^C) compared to C3 plants (approximately −13‰ vs. −28‰, respectively) (Fry 2006). In marine systems, high *δ* ^13^C values are characteristic of benthic production, while lower values are associated with pelagic producers (Barros et al. 2010). These differences in *δ* ^13^C values are passed up the food web to consumers, providing a quantifiable estimate of habitat and resource use. In contrast, nitrogen isotope ratios (^15^N/^14^N) increase at each trophic step with top predators having higher nitrogen isotope values (*δ* ^15^N) compared to lower trophic levels (Fry 2006). Thus, *δ* ^15^N values can characterize an important component of the bionomic niche of a population (Layman et al. 2007; Newsome et al. 2010). To date, the vast majority of isotopic studies attempting to characterize trophic niche space use only C and N stable isotopes (Newsome et al. 2010; Layman et al. 2011). Yet, other isotopes quantify important components of niche space. The stable isotopes of S, O, H, Sr, and Pb have been used in ecological studies to examine foraging latitude, natal watersheds, marine food web reliance, ontogenetic diet shifts, and migration (reviewed in Newsome et al. 2007; Ostrom et al. 2014; Rossman et al. 2015). However, until recently methodological approaches for quantitatively defining isotopic niche space were limited to the incorporation of only two isotopes (Swanson et al. 2015).

A number of quantitative tools have been developed to estimate niche space across isotopic variation (reviewed in Layman et al. 2011). Historically, most stable isotope analyses used ANOVAs to compare sample means for each isotope individually, but this approach failed to adequately characterize variation in stable isotope values. Layman et al. (2007) proposed the use of convex hull area – the minimum area that contains every *δ* ^13^C‐*δ* ^15^N value in a sample – (among other metrics) to quantify isotopic niche space. Turner et al. (2010) expanded Layman's approach to produce a method to test for differences in niche position by calculating the distance between centroid locations of two populations within the convex hull. Although convex hulls provide a measure of niche width across scenopoetic and bionomic niche axes, the metric is sensitive to sample size (Jackson et al. 2011). Convex hulls also do not account for uncertainty associated with sampling because only a point estimate is produced. Jackson et al. (2011) suggested a more rigorous approach to measure isotopic niche width using a multivariate, ellipse‐based metric (see the SIBER, “Stable Isotope Bayesian Ellipses in R,” package in R). The size and orientation of the ellipse depicts variability in a population's resource use across *δ* ^13^C and *δ* ^15^N axes. The orientation of the ellipse is determined by the axis of maximum variation in *δ* ^13^C and *δ* ^15^N values (e.g., the first principal component of a PCA or a regression line). More variation in the data results in a larger ellipse. The shape of the ellipse changes based on the strength and sign (positive or negative) of the correlation between the two isotopes. For example, a population in which *δ* ^13^C and *δ* ^15^N value are uncorrelated will result in a near‐circular ellipse (if variances in *δ* ^13^C and *δ* ^15^N are similar), while highly correlated isotope values will produce long, thin ellipses. Standard ellipse area offers a statistically robust means of comparing differences in isotopic niche widths across populations (Jackson et al. 2011). Swanson et al. (2015) advanced ellipse‐based models further by allowing for the inclusion of more than two niche dimensions and presenting a probabilistic framework for calculating niche overlap. Previously, the area of niche overlap could only be calculated as a point estimate. Additionally, the associated R package “nicheROVER” (Lysy et al. 2014) provides a means for researchers unfamiliar with statistical coding a means of preforming complex niche modeling.

Incorporating additional stable isotopes into niche estimates yields a better understanding of food web dynamics and a model, which more accurately captures ecosystem complexities (Swanson et al. 2015). For instance, in marine systems, *δ* ^34^S values can differentiate organisms that forage in shallow nearshore environments from those that forage offshore (Barros et al. 2010), and *δ*D values may distinguish between invertebrates and teleost fish consumption in seabirds (Ostrom et al. 2014). Historically, the inclusion of only two stable isotopes was not problematic as most isotope ratio mass spectrometers were set up for specifically measuring one or two isotopes at a time. Newer instrumentation, however, commonly measures C, H, O, N, and S stable isotopes – often simultaneously – increasing the need for more complex niche models.

We provide a unified method to quantify three‐dimensional isotopic niche space using a transparent user‐defined model code, which can easily be changed and adapted to suit an investigator's specific research needs. Our approach estimates a population's isotopic niche by calculating its standard ellipsoid volume (SEV) and centroid location (CL) while explicitly calculating uncertainty in these measurements allowing ecologists to compare both niche width (SEV) and location (CL) to assess biologically meaningful differences in niche space across groups, populations, species, or communities. We demonstrate the accuracy, precision, and required sample sizes of multidimensional niche modeling with a simulation study across a spectrum of hypothetical data. We also calculate the SEV, CL, and isotopic niche shape for three populations of parapatric bottlenose dolphins off the Florida Gulf Coast, revealing significant differences in trophic niche that were undetectable using standard two‐dimensional approaches.

### The model

The modeling framework is analogous to that of Swanson et al. (2015) and represents the three‐dimensional extension of Jackson et al. (2011). Standard ellipsoid volumes used to estimate isotopic niche are constructed using (1) the three mean population‐level isotope values (***μ** *= *μ*_x_, *μ*_y,_ *μ*_z_) and (2) the associated 3 × 3 covariance matrix (**Σ**) in which diagonal elements represent intrapopulation isotope variances and off diagonal elements are pairwise covariances between stable isotopes. We assume that each data point, three isotope values for individual *i* (denoted **y**_*i*_), derives from a multivariate normal distribution,

$$y_{i}∼\text{MVN}\left(\mu ,\Sigma \right),$$

allowing for the estimation of the population‐level parameters ***μ*** and **Σ** from the sample data. The centroid location (CL) of the ellipsoid is given by the vector ***μ***. The ellipsoid shape varies based on values of **Σ**, with the semimajor axis oriented along the plane of maximum variation in the population and the two semiminor axes orthogonal to the semimajor axis and one another. The lengths of the semimajor and semiminor axes are calculated using the eigenvalues of **Σ**. The semimajor axis, a, is derived from the first eigenvalue ($a=\sqrt{\lambda_{1}}$), and the semiminor axes b and c are similarly calculated using the second and third eigenvalues $\left(b=\sqrt{\lambda_{2}},c=\sqrt{\lambda_{3}}\right)$. Ellipsoid volume is:

$$\text{SEV}=\frac{4}{3}\pi abc.$$

We estimate ***μ*** and **Σ** using a Bayesian approach. The Bayesian approach produces a full posterior probability distribution for all parameters including CL and SEV (as derived parameters). This allows us to calculate moments of the posterior distribution (mean, median, and mode) as well as uncertainty in estimates of CL, shape, and SEV in *δ*‐space (i.e., a 95% credible interval). Additionally, credible intervals (CI) for parameter estimates are asymptotically unbiased at small sample sizes (Gelman and Hill 2007), a desirable property as sample sizes are often small in stable isotope studies. Bayesian analysis requires specification of a prior distribution for all parameters, presenting the opportunity to use known information (e.g., from other studies) or left purposefully uninformative (as we do for all analyses in this paper).

### Simulation study

We developed a simulation study to explore the minimum sample size necessary for unbiased estimation of a population's CL and SEV as well as the minimum sample size necessary to detect differences between populations with differing SEVs and CLs. We chose four hypothetical populations spanning a range of realistic SEV values for stable isotope studies (Fry 2006). The four simulated populations had true SEVs of 5‰^3^ (population one), 7.5‰^3^ (population two), 10‰^3^ (population three), and 20‰^3^ (population four) and true centroids of (0,0,0), (1,1,1), (2,2,2), and (3,3,3), respectively. The absolute values of the centroids are not important; rather, interest lies in our ability to estimate their relative differences from one another. Thus, our intention is to determine the number of samples necessary to detect the differences between population one and each of the three other populations, the relative locations of which are increasingly disparate in *δ*‐space. We generated 1000 datasets for each sample size from *n* = 6–100 for each of the four populations. To do this, we developed a function to produce random covariance matrices for a given SEV value and then used the generated covariance matrices and the true mean centroid vector to produce the datasets with the function rmvnorm in R (R Core Development Team 2013). Each dataset was analyzed using Markov chain Monte Carlo (MCMC) sampling in programs R and JAGS to estimate posterior distributions for both ***μ*** and **Σ** (Plummer 2003). We used vague normal priors for values of ***μ***, centered at zero with variances of 1000. The inverse‐Wishart prior was used for **Σ** (a diagonal 3 × 3 matrix with values of 3 and 4 degrees of freedom), which is the conjugate prior of the multivariate normal distribution (Gelman and Hill 2007).

We ran each simulation for 4000 iterations and discarded the first 1000 as a burn‐in for each of the 1000 datasets at every sample size 6–100, to produce nearly 100,000 independent runs. Initial runs indicated adequate model convergence. We estimated the probability that populations two, three, and four had larger SEV than that of population one by calculating the proportion of MCMC iterations in which SEV_[pop. 2, pop. 3, or pop. 4]_ > SEV_pop. 1_ for each pairwise run of the model. We calculated distances between population centroids in *δ*‐space according to Euclidean distance:

$$D_{\mu_{1},\mu_{2}}=\sqrt{{\left(\mu_{x_{2}}-\mu_{x_{1}}\right)}^{2}+{\left(\mu_{y_{2}}-\mu_{y_{1}}\right)}^{2}+{\left(\mu_{z_{2}}-\mu_{z_{1}}\right)}^{2}}.$$

$D_{\mu_{1},\mu_{2}}$ is always positive, which presents a challenge in determining whether there are statistically or biologically significant differences in CLs among populations because the posterior interval of *D* never overlaps zero, even for two ellipsoids with the same ***μ*** values. To account for this, we divided the posterior estimates of ***μ*** into null (*n*) and test (*t*) distributions of equal size. We then calculated the probability that the distance between two centroid values in the test distributions ($\mu_{1_{t}}$ and $\mu_{2_{t}}$) was greater than the distance between the test and null distributions for ***μ***_1_ and ***μ***_2_, respectively:

$$\begin{array}{cc} P[D_{\mu_{1},\mu_{2}}>0]= & \sum (D\left[\mu_{1_{t}},\mu_{2_{t}}\right]-D\left[\mu_{1_{t}},\mu_{1_{n}}\right]\\ & \frac{-D[\mu_{2_{t}},\mu_{2_{n}}]>0)}{\text{total number of posterior estimates}}\\ & \text{in null or test distributions} \end{array}$$

This produces a conservative estimate of the probability two CL are different, which is useful for hypothesis testing. Appendix S1, Panel S1 contains the complete model code for the simulation study.

We developed a method to calculate the geometric area of niche overlap using naïve numeric integration. The area of overlap is calculated and scaled with respect to the SEV of the target population as follows:

$$\%\;{\text{overlap}}_{1,2}=\frac{A_{1,2}}{{\text{SEV}}_{1}}$$

where the percent of niche overlap between populations one and two with respect to the niche volume of population one is the volume of overlap between populations one and two divided by the SEV for population one. This calculation is preformed for each estimate of the posterior distribution to create an estimate of the certainty in the percent overlap estimate.

While we provide code to calculate percent overlap between ellipsoids (Appendix S1), it was not included as part of the simulation study. The area of overlap is derived from estimates of ***μ*** and **Σ** thus if the model can estimate those parameters accurately including area of overlap in the simulation study is superfluous and computationally taxing.

## Results

**Figure 1:**
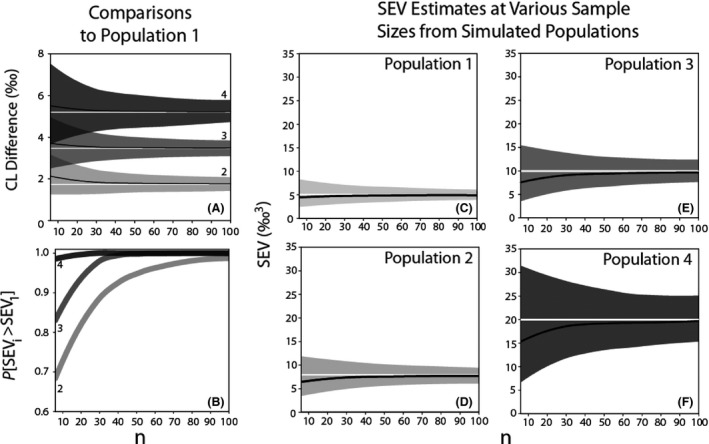
(A) Median estimated (black lines) and true (white lines) differences in the centroid location (CL) between population one and populations two through four (corresponding to number on right side of panel). Medians are calculated using 1000 simulated datasets at each sample size *n* = 6 through *n* = 100. The shaded gray areas show the 95% CI. (B) Median probability of correctly estimating that populations – two through four (number on left side of panel) had a larger SEV compared to population one for sample sizes ranging from 6–100. (C–F) Median estimated (black lines) and true (white lines) standard ellipsoid volumes (SEV) for four populations with progressively increasing isotopic niche size. Shaded gray areas show the 95% CIs.

Results indicate the model produces accurate estimates of CL at all sample sizes. Differences in CL between populations also are accurately detected by the model (Fig. 1A). The model had ≥95% probability in detecting the true difference in CL values for 85% of datasets with only six samples per population when the true distance in CL was only ~1.7‰ (comparison of population one to population two). At larger differences in CL and at larger sample sizes, the average probability of detecting differences between two populations is nearperfect. Absolute estimates of CL differences are nearly unbiased at even the smallest sample sizes for all populations, showing only a slight overestimation when sample sizes are small (Fig. 1A).

The model successfully detects the larger SEV in pairwise comparisons of populations across the complete range of sample sizes tested in the majority of simulations (Fig. 1B). When the differences in SEV between populations are relatively large (population four – population one = 15‰^3^), the model correctly identifies the population with a larger trophic niche with near‐perfect certainty with a sample size of only six individuals per population (median probability = 0.98). When the differences in SEV are small (population two – population one = 2.5‰^3^), our model correctly identifies the population with the larger SEV in approximately 70% of runs with a sample size of only six individuals per population. This is particularly remarkable considering that the difference in SEV between populations one and two equates to an approximate difference of 0.22‰ in average intrapopulation standard deviation of each isotope (average standard deviation per isotope for population 1: 1.06‰ vs. population 2: 1.28‰). However, when two population's SEV values are this similar, power is low. Thus, large sample sizes are necessary for high certainty of the differences (e.g., 90% certainty requires a minimum of ~40 individuals per population when the true difference in SEV is less than 5‰^3^) (Appendix S1, Figure S1).

The 95% CI for SEV contains the true value for each population at every sample size in all simulations. However, moments from the SEV posterior distribution (mean, median, mode) are underestimated when sample size is small, particularly for populations with large SEV values (Fig. 1C–F). For example, the median SEV estimates in our simulation study did not approach the true value until *n *>* *40 in the population with the largest SEV (e.g., population four with 20‰^3^). Although SEV may be underestimated at small sample sizes, this is not necessarily problematic because the model identifies differences in SEV sizes between populations fairly accurately (e.g., Fig. 1B). Thus, the model can be safely used for hypothesis testing on both relative isotopic niche location and size, particularly once sample sizes are above 20 per population or differences in SEV are greater than 5‰.

### Application: niche variation in parapatric bottlenose dolphin populations

**Figure 2:**
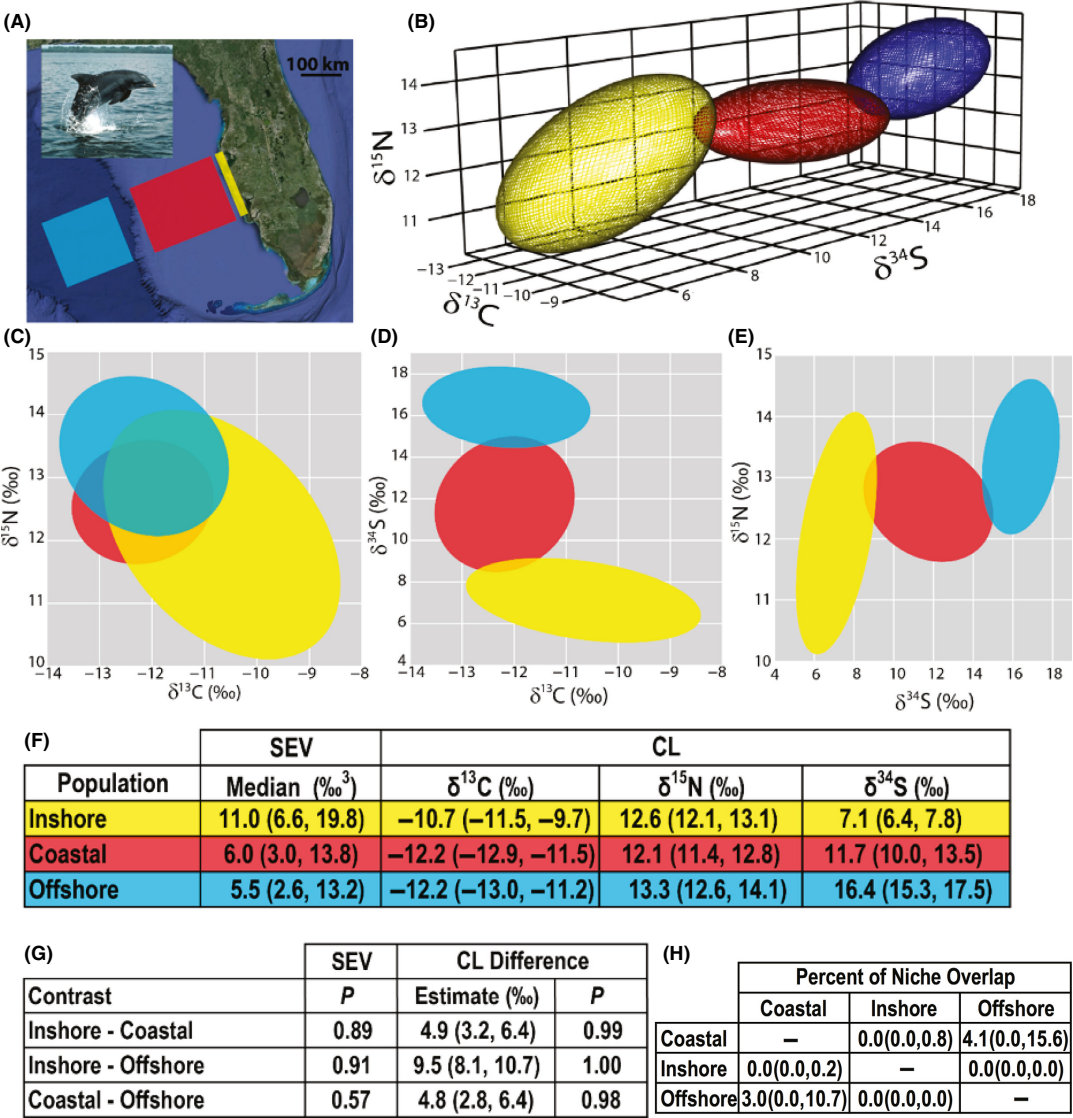
(A) Bottlenose dolphin and approximate ranges for inshore (yellow), coastal (red), and offshore (blue) populations inhabiting the Gulf coast off western Florida. Ranges for offshore and coastal dolphins are illustrative only, as little is known about their exact movements. (B) The estimated isotopic niches for the inshore (yellow), coastal (red), and offshore (blue) populations graphed in three dimensions. (C–E) Three‐dimensional ellipsoids compressed to three‐two‐dimensional plots. (F) Estimated standard ellipsoid volume (SEV) and centroid location (CL) values (median and 95% CI range) for each of the three populations. (G) The SEV column shows the probability that the first listed population has a greater SEV value than the second listed population. The second column shows the estimated differences in CL in pairwise comparisons between populations and the final column lists the probability that the two population have different CL values. (H) The area of overlap between each population divided by the SEV of the population listed in the row to produce the percent of niche overlap (median and 95% CI range).

Common bottlenose dolphins (*Tursiops truncatus*) possess a nearly pan‐global distribution, with few natural barriers preventing dispersion and gene flow. Yet, populations within close geographic proximity often show considerable genetic distinction (Wells 2014) because differences in habitat use or prey type can facilitate segregation in social interactions (Natoli et al. 2005). Such segregation can be identified via stable isotope analysis. For example, carbon isotope values vary between benthic primary production (i.e., seagrass) and phytoplankton‐dominated, pelagic habitats; nitrogen isotope values are positively correlated with trophic level; and sulfur isotope values increase with salinity (Newsome et al. 2010). Together, these three stable isotopes provide detailed information on both scenopoetic and bionomic niche axes by which we can test for niche segregation among three putative bottlenose dolphin populations inhabiting the Gulf of Mexico (Fig. 2A). The first population (inshore) is comprised of resident dolphins that predominantly utilize shallow waters, often between the mainland and barrier islands. The second group (coastal) contains dolphins that are hypothesized to use shallow waters beyond the barrier islands. The third population (offshore) encompasses dolphins that use waters beyond the continental shelf and have been known to differ morphologically from the inshore and coastal populations.

Tooth samples used for isotopic analysis were collected from dead, stranded dolphins recovered near Sarasota Bay, Florida or Charlotte Harbor, Florida. The dataset contains 18 inshore, nine coastal, and eight offshore dolphins collected between 1977 and 2007. Tooth samples were homogenized, demineralized, and lipids extracted before analysis on an elemental analyzer interfaced to an isotope ratio mass spectrometer. Additional details on these bottlenose dolphin populations and the data collection process are in Barros et al. (2010). We ran our model separately for each population using the carbon, nitrogen, and sulfur isotope data. We used the priors and MCMC settings described in the simulation study.

Our results indicate the three dolphin populations occupy different trophic niches (Fig. 2B–E). The inshore population has a much larger SEV compared to both coastal (0.89 probability that SEV_inshore_ > SEV_coastal_) and offshore (0.91 probability that SEV_inshore_ > SEV_offshore_) populations, suggesting that the inshore population possesses the widest isotopic niche. Coastal and offshore dolphin populations possess similar isotopic niche widths (e.g., SEV values). The dolphin populations differ in niche position (CL), with the inshore population being most disparate from the offshore population. Coastal dolphins occupy an intermediate niche between and equidistant from inshore and offshore dolphins. CLs for all three populations had high probabilities of being different in pairwise comparisons, indicating that these populations occupy unique isotopic niches and thus use the waters off West Central Florida in profoundly different manners (Fig. 2F–G). Percent overlap also showed distinction between the three populations. The maximum percent overlap occurred between offshore and coastal populations and has a 95% probability of being below 15.6% and 10.1% of each populations niche width, respectively (Fig. 2H).

The three Gulf of Mexico bottlenose dolphin populations show dramatic isotopic niche differentiation (Fig. 2B and G). However, this finding would have been overlooked if not for the inclusion of *δ* ^34^S. Niche space for these three populations erroneously appears overlapping in *δ* ^13^C–*δ* ^15^N space, with coastal and offshore populations seemingly occupying nearly identical niches and with more variation existing in the inshore population (Fig. 2C). Niche overlap among the three populations dramatically decreases when sulfur isotopes are included in the analysis (Fig. 2D and E), revealing that each of these populations possesses a unique niche, particularly with regard to salinity. Inshore dolphins likely possess the largest isotopic niche because estuaries are structurally complex, with a diverse array of prey species and habitats (Wells 2014; Rossman et al. 2015). Conversely, offshore habitats tend to be structurally homogenous, with sporadic patches of dense prey. The foraging skills that make an animal successful in an estuary likely do not transfer well to offshore environments. Additionally, social learning from conspecifics and matrilineal transmission of foraging skills play a role in the development of bottlenose dolphins (Wells 2014; Rossman et al. 2015). These factors may have resulted in habitat and prey‐specific foraging specialization to produce the observed niche segregation, likely contributing to the genetic differentiation among bottlenose dolphin populations.

## Discussion

Ellipsoid‐based niche model are a relatively new quantitative tool to study population‐level trophic niche space in three isotopic dimensions. Researchers were previously required to use ad hoc tests which utilize fundamentally different statistical paradigms, such as testing for difference in CL via the methods of Turner et al. (2010) while constructing Bayesian ellipse to compare isotopic niche size (Jackson et al. 2011). Our code (Appendix S1) provides a single unified framework to estimate and test for difference in niche size, shape, location, and overlap providing a transparent and parsimonious statistical approach.

Isotopic studies that aim to quantify scenopoetic and bionomic axes of niche space have historically been limited to two isotopes. As analysis of H, O, and S stable isotopes become more readily accessible, isotopic modeling must also expand. Narrowing the scope to only C and N isotopes limits our understanding of niche space and can lead to fundamentally misleading conclusions about niche overlap, as demonstrated with our example of bottlenose dolphins in the Gulf of Mexico. Swanson et al. have provided the stable isotope ecology community a readily accessible package in R, which allows those not familiar advanced statistical methods access to three‐dimensional niche modeling. We present an alternative for those who want full control and customization of their statistical models. Our three‐dimensional isotopic model code is extremely flexible, providing an easily adaptable framework (Appendix S1) that can be used for niche hypothesis testing among populations and easily adapted to suit investigators' specific needs. For instance, our code could be adapted to treat individuals within a population as random variables derived from some mean and variance value for the population. In this process, within‐individual variation is parsed between individual variation in a statistically robust framework. The code can also be used to test whether there are correlations in niche axes, such as whether foraging in a particular habitat is associated with feeding at a higher trophic level, by examining whether the CIs of the covariance terms in **Σ** overlap zero.

Expanding our model code beyond three dimensions can easily be accomplished with modifications to the supplied Appendix (S1); however, at this time we caution users against moving beyond three niche axes. We have extensively tested our three‐dimensional model using a simulation study to ensure a population's isotopic niches parameters can be recovered precisely and unbiasedly – something that Swanson et al. has not yet performed for *n* dimensions. While theoretically useful, the *n*‐dimensional niche modeling approach presented by Swanson et al. (2015) has not been properly vetted and thus researchers are not aware of potential biases, necessary sample size, or power. We provide detailed results on the ability of the model to distinguish isotopic niche space between populations with various sample sizes and on the bias trade‐off associated with prior choice for the multivariate normal distribution in three dimensions.

Despite the quantitative advances presented by stable isotope niche modeling, the fundamental biogeochemistry in a system and the assumptions of a given model should be considered when using stable isotopes to make inferences about niche space. Not all variation in isotope values is indicative of differences in resource use (see caveats in Layman et al. 2011). Factors not related to resource use could conflate estimates of SEV and bias CL locations if the physiology of the study organism and biogeochemistry of the system are not well understood. Additionally, increasing the dimensionality of the model with additional isotopes does not circumvent the need to have sufficient isotopic variation to differentiate groups. Researchers should consider how the phototrophic base of the food web, biogeochemistry of the ecosystem, rate of tissue turnover, and trophic discrimination, may impact isotope values and thus model results (Flaherty and Ben‐David 2010; Layman et al. 2011; Phillips et al. 2014). An important assumption in our model is that isotope values within a sample can be described using a multivariate normal distribution. The multivariate normal allows for a wide range of covariance relationships among variables, which renders it particularly useful for stable isotope analyses. The vast majority of studies use a series of independent normal distributions to model isotopes separately, which can constrain results.

Bayesian methods provide a useful approach to analyzing stable isotope models because they produce a posterior distribution for each model parameter, which can be then used to derive other quantities of interest (e.g., SEV). This readily allows for complex hypothesis testing. Bayesian methods require specification of prior distributions for all model parameters (***μ*** and **Σ** in this case). We used the inverse‐Wishart prior for **Σ** because it is the conjugate prior for the multivariate normal and thus a natural choice. However, the Wishart distribution introduces information by specifying a specific structure among covariances. As a result, the prior can introduce bias at small sample sizes and likely accounts for the observed underestimation of SEV when sample size is low and/or SEV is large (Swanson et al. 2015). When the variance components of **Σ** are large (resulting in a large SEV; e.g., population 4), the inverse‐Wishart overestimates correlations between stable isotopes (Alvarez 2014), resulting in thinner, more oblong ellipsoids and smaller SEV estimates (e.g., Fig. 1F). This bias decreases as sample size increases because information in the data is sufficient to overwhelm the prior. In early iterations of model development, we explored the possibility of using individual priors on each term in **Σ**, (Appendix S2, Panel S1). The model with individual priors on **Σ** produced unbiased SEV estimates at both small sample sizes and high variance (Appendix S2, Figure S1). However, the trade‐off is extremely large CIs on parameter estimates and results that are frequently further from the true SEV value than those produced using the inverse‐Wishart based model (Appendix S2, Figure S2). We thus suggest using the inverse‐Wishart even though it produces slightly biased estimates of SEV at small sample sizes because it is capable of more accurately and precisely distinguishing differences in SEVs among populations. For alternative priors on the covariance matrix see Swanson et al. (2015, appendix S3).

### Guidelines

When variance within population is small ellipsoid‐based niche models preform well showing little to no bias in SEV and CL with as few as six samples. As variance in the niche axes increase more samples are needed. When SEV = 20 (which roughly equates to a standard deviation of 2.5 across the three niche axes) over 30 samples are need for an unbiased estimate, although we recognize this is difficult to determine *a priori*. Thus, researchers should be conservative when drawing conclusions related to differences in niche volume which variance is high and sample size is low, particularly when the niche metrics consists of three or more dimensions.

Stable isotopes provide a powerful tool to delineate otherwise cryptic foraging behavior across a wide array of ecosystems and taxa. They are often the only means to study foraging habits of animals that use large geographic areas, capture and consume prey in difficult to observe habitats, or are found deceased/are extinct. While estimates of isotopic niche space cannot provide high precision on dietary information, they are among the few methods capable of quantitatively testing variation in niche. By expanding niche‐based metrics from two to three dimensions, isotope‐based niche estimates are more reflective of how animals use their environments in the real world than traditional methodologies. Multivariate modeling framework can accompany other forms of data (i.e., GIS or morphometric data) to enhance analyses of species' niches (Rossman et al. 2015) and allow ecologists to ask ever more complex questions.

## Conflict of Interest

None declared.

## Supporting information


**Appendix S1.** Additional details on the simulation study.

**Appendix S2.** Standard ellipsoid model using individual priors on terms within the covariance matrix, Σ.
