# Supplementary material for: Beyond carbon and nitrogen: guidelines for estimating three‐dimensional isotopic niche space
Source: Ecol Evol. 2016 Mar 9;6(8):2405–13. doi: 10.1002/ece3.2013 (PMC4834325; doi:10.1002/ece3.2013)

**Appendix A: Additional details on the simulation study**

This appendix includes the R and JAGS code used to run the simulation study (Panel A1) and additional simulation study results (Figure A1).

**Panel A1.** The R and JAGS code used to simulate CL and SEV for four different populations with various true CL and SEV values. We assigned each population a true CL and SEV values. The true SEV value is then used to calculate a semi-random covariance matrix, **Σ**, with the function “getcov(SEV)”. The true CL was used as the mean vector, **µ**, for each ellipsoid. For illustrative purposes, the code below generates 20 observations for each population from a multivariate normal distribution with the mean vector **µ** and the simulated covariance matrix **Σ**. SEV was calculated as a derived quantity based on posterior estimates of **Σ** after the JAGS model run was completed in R. The code used for hypothesis testing for differences in CL, SEV, and distance between centroids is also included.

library(MASS) # for mvrnorm

library(jagsUI) # to run jags

#Function to calcuate SEV

SEV.function<-function(sigma) {

if (eigen(sigma)$values[3] > 0)

{

axises<-sqrt(eigen(sigma)$values)

}

else {

axises<-NA

}

out<-list()

out$a<-axises[1]

out$b<-axises[2]

out$c<-axises[3]

out$SEV<-prod(axises,pi,4/3)

return(out$SEV)

}

overlap<-function(mu1, mu2, sigma1, sigma2){

axises<-sqrt(eigen(sigma1)$values)

}

#create a matrix of group combinations for pairwise comparisons

pairwise<-function(ngroups){

groups<-seq(1,ngroups)

max.compare<-((ngroups-1)*ngroups)/2

i=0

counter=ngroups

pairs<-c(0,0)

while (counter>0){

i=1+i

counter=counter-1

if (counter!=0){

temp1<-rep(groups[i],counter)

temp2<-seq(i+1,ngroups)

pairs<-rbind(pairs,cbind(temp1,temp2))

if (i==1){

pairs<-pairs[-1,]

}

}}

colnames(pairs)<-c('group1','group2')

return(pairs)

}

#Function to generate a random covariance matrix based on given SEV value

getcov<-function(SEV){

options(warn=-1)

Sigma<-matrix(rep(NA,9),ncol=3)

while(is.na(Sigma[1,1]==TRUE)){

if (((3*SEV)/(4*pi))^2/3 < 1){

lambda1<-runif(1,1,((3*SEV)/(4*pi))^2)

}else {

lambda1<-runif(1,((3*SEV)/(4*pi))^2/3,((3*SEV)/(4*pi))^2)

}

lambda2<-runif(1,(((3*SEV)/(4*pi))^2)/lambda1,((3*SEV)/(4*pi)))

lambda3<-(((3*SEV)/(4*pi))^2)/(lambda1*lambda2)

u<-diag(c(lambda1,lambda2,lambda3))

Q<-qr.Q(qr(runif(3,-0.8,.8)),complete=TRUE)

Sigma <- Q %*% u %*% t(Q)

}

options(warn=0)

return(Sigma)

}

#Calculate euclidian distance between two 3 demensional points

distance<-function(a,b){

l<-sqrt((a[,1]-b[,1])^2+(a[,2]-b[,2])^2+(a[,3]-b[,3])^2)

return(l)

}

#calculate the mode of a posterior distrobution

mode <- function(s) {

d <- density(s,na.rm=TRUE)

d$x[which.max(d$y)]

}

#functions to calculate overlap

# This function calculates area of SEV overlap via naive for-loop numeric integration of one.

# ellipsoid step function (1 when MVNdist2<1) over the other ellipsoid, done as a 3D Riemann

# sum over little cubes of variable size. The user specifies the variable "HowFine", and

# this determines cube size on a log scale. Specifically, the cube width is equal to "OneStep",

# which is defined by OneStep <- 10^(-HowFine). This piece of code makes a big list to index

# all the little cubical regions inside the ellipsoid E1 at this resolution, counts how many

# of them are inside E2, and then multiplies this number by the volume of each little cube,

# which is CubeVol <- OneStep^3, to produce the approximate overlap volume.

CubeCount3D <- function(mu1,mu2,S1,S2,HowFine=1.0,ShowProgress=FALSE){

# set-up stuff:

MVNdist <- function(mu,S){function(x){t(x-mu)%*%solve(S)%*%(x-mu)}}

OneStep <- 10^(-HowFine);

CubeVol <- OneStep^3;

CubeCt <- 0;

A1 <- eigen(S1)$vect

dD1 <- eigen(S1)$val

MVNdist2 <- MVNdist(mu2,S2);

# the loops:

zlist <- seq(from=0,to=dD1[3]^.5,by=OneStep)

zlist <- if(length(zlist)>1){c(rev(-zlist),zlist[2:length(zlist)])}

for(Z in zlist){

yBd <- (dD1[2]*(1-Z^2/dD1[3]))^.5

ylist <- seq(from=0,to=yBd,by=OneStep)

ylist <- if(length(ylist)>1){c(rev(-ylist),ylist[2:length(ylist)])}

for(Y in ylist){

xBd <- (dD1[1]*(1-Y^2/dD1[2]-Z^2/dD1[3]))^.5

xlist <- seq(from=0,to=xBd,by=OneStep)

xlist <- if(length(xlist)>1){c(rev(-xlist),xlist[2:length(xlist)])}

for(X in xlist){

if(MVNdist2(A1%*%c(X,Y,Z)+mu1)<1){CubeCt=CubeCt+1}

}

}

# This will show a "progress report" in the console window as this script is running:

# (0=start, 1=end)

if(ShowProgress){print((.5*(Z+dD1[3]^.5))/dD1[3]^.5)}

}

return(CubeCt*CubeVol)

}

##############################################################################

#Jags mode

##############################################################################

sink("ellipsoid_example.txt")

cat("

model{

#priors

for (k in 1:ngroups){

mu[k,1] ~ dnorm(0,0.001)

mu[k,2] ~ dnorm(0,0.001)

mu[k,3] ~ dnorm(0,0.001)

prec[1:3,1:3,k] ~ dwish(S3,4)

cov[1:3,1:3,k] <- inverse(prec[1:3,1:3,k])

#likelihood

for (i in 1:maxn){

y[i,1:3,k] ~ dmnorm(mu[k,],prec[,,k])

}#i

}

}

",fill = TRUE)

sink()

##############################################################################

# Generate Data

##############################################################################

# Data should be supplied as a four column table, the fist column is a grouping

# variable which can be catagorical or numeric

ngroups<-4

n<-20

TrueSEV<-c(5,7.5,10,20)

S<-array(NA, dim=c(3,3,ngroups))

for (i in 1:ngroups){

S[,,i]<-getcov(TrueSEV[i])

}

TrueMu<-rbind(c(0,0,0),c(1,1,1),c(2,2,2),c(3,3,3))

thedata<-array(NA, dim=c(n,4,ngroups))

for (i in 1:ngroups){

thedata[,,i]<-cbind(rep(paste("group",i),n),mvrnorm(n,TrueMu[i,],S[,,i]))

}

thedata<-as.data.frame(apply(thedata,2,c))

colnames(thedata)<-c("groups","isotope1","isotope2","isotope3")

##############################################################################

# Preparing the data

##############################################################################

#generate list names of each group

groupnames<-levels(as.factor(thedata[,1]))

#generate list of isotope names and number of isotopes

isotope_names<-colnames(thedata)[2:4]

isotopes<-3

#convert group names to numeric factors

thedata[,1]<-as.numeric(as.factor(thedata[,1]))

ngroups<-max(thedata[,1])

#find the groups with the largest n and the n per group

nstart<-c(NA)

nend<-c(NA)

for (i in 1:ngroups){

nstart[i]<-min(as.numeric(rownames(thedata[thedata[,1]==i,])))

nend[i]<-max(as.numeric(rownames(thedata[thedata[,1]==i,])))

}

maxn<-max(nend+1-nstart)

npergroup<-nend+1-nstart

# create an array where nrow = the n of the largest group, for groups with smaller n's

# some rows will have NA's to account for uneven replication

y<-array(NA,dim=c(maxn,3,ngroups), dimnames=c(NA,isotope_names,groupnames))

for (i in 1:ngroups){

y[1:nrow(thedata[thedata[,1]==i,2:4]),,i]<-as.matrix(thedata[thedata[,1]==i,2:4])

}

#ensure y is a completely numeric array

y<-array(as.numeric(y),dim=dim(y))

#####################################

#package the data

#####################################

#objects passed to jags

data=list(y=y,ngroups=ngroups, maxn=maxn, S3=(diag(3)*3))

#initial values

mu0<-matrix(NA, nrow=ngroups, ncol=3)

prec0<-array(0,dim=c(3,3,ngroups))

for (k in 1:ngroups){

mu0[k,]<-mvrnorm(1,c(0,0,0),diag(3)*100)

prec0[,,k]<-getcov(runif(1,5,30))

}

inits=function(){list( mu=mu0, prec=prec0)}

# parameters to moniter

params<-c("mu","cov")

# MCMC settings

nt <- 1

nb <- 1000

nc <- 3

na<-1000

ni <- 5000

#################################################

# Run Jags

################################################

out <- jags(data = data,

inits = inits,

parameters.to.save = params,

model.file = 'ellipsoid_example.txt',

n.chains = nc,

n.adapt = na,

n.iter = ni,

n.burnin = nb,

n.thin = nt)

#plot(out)

summary(out,digits=2)

#################################################

# Calulate SEV

###############################################

# a list to index pairwise comparisons

pl<-cbind(sort(rep(seq(1:ngroups),ngroups)),rep(seq(1:ngroups),ngroups))

# probabilities to posterior distrobutions

probs<-c(0.025,0.5,0.975)

# Calculate an estimate of SEV from each posterior estimate of sigma and sumarize

SEV<-matrix(NA,ncol=ngroups,nrow=nrow(out$sims.list$cov))

SEV.summary<-matrix(NA,nrow=ngroups,ncol=3+length(probs))

for (k in 1:ngroups){

SEV[,k]<-apply(out$sims.list$cov[,,,k],1,SEV.function)

SEV.summary[k,]<-c(nend[k]+1-nstart[k],quantile(SEV[,k],probs=probs),mode(SEV[,k]), mean(SEV[,k]))

}

rownames(SEV.summary)<-groupnames

colnames(SEV.summary)<-c("n",paste(probs,"%",sep=""),"mode","mean")

#################################################

# SEV Pairwise comparison

###############################################

#Compare each posterior SEV estimate to those of other groups

SEV.diff<-array(NA,dim=c(ngroups,ngroups,nrow(SEV)))

for(i in 1:(ngroups*ngroups)){

SEV.diff[pl[i,1],pl[i,2],]<-SEV[,pl[i,1]]-SEV[,pl[i,2]]

}

p.table<-matrix(NA,nrow=ngroups,ncol=ngroups)

colnames(p.table)<-paste(">",groupnames, sep="")

rownames(p.table)<-groupnames

for (i in 1:(ngroups*ngroups)){

p.table[pl[i,1],pl[i,2]]<-sum(SEV.diff[pl[i,1],pl[i,2],]>0)/nrow(SEV)

}

#################################################

#Pairwise comparison centroid

###############################################

#compare posterior estimates of mu (isotope mean values) to those other groups

mu.diff<-array(NA, dim=c(nrow(out$sims.list$mu),3,ngroups*ngroups))

for (i in 1:(ngroups*ngroups)){

mu.diff[,,i]<-out$sims.list$mu[,pl[i,1],]-out$sims.list$mu[,pl[i,2],]

}

mu.p<-rep(list(matrix(NA,ncol=ngroups,nrow=ngroups)),3)

for (k in 1:3){

for(i in 1:(ngroups*ngroups)){

mu.p[[k]][pl[i,1],pl[i,2]]<-sum(mu.diff[,k,i]>0)/nrow(out$sims.list$mu)

}}

for (k in 1:3){

rownames(mu.p[[k]])<-groupnames

colnames(mu.p[[k]])<-paste(">",groupnames, sep="")

}

names(mu.p)<-isotope_names

print(mu.p,digits=2)

#################################################

# Distance between centroids

###############################################

# Divide posterior estimates of mu into test and null distrobutions

null_mu<-array(out$sims.list$mu[seq(1,nrow(out$sims.list$mu),by=2),,],dim=c(nrow(out$sims.list$mu)/2,ngroups,3))

test_mu<-array(out$sims.list$mu[seq(2,nrow(out$sims.list$mu),by=2),,],dim=c(nrow(out$sims.list$mu)/2,ngroups,3))

dist.diff<-array(NA, dim=c(ngroups,ngroups,nrow(out$sims.list$mu)/2))

dist.diff.test<-array(NA, dim=c(ngroups,ngroups,nrow(out$sims.list$mu)/2))

#Calculate distance estites for each posterior of mu and a distanace measure adjusted based on the

# null distrobution

for (i in 1:(ngroups*ngroups)){

dist.diff[pl[i,1],pl[i,2],]<-distance(test_mu[,pl[i,1],],test_mu[,pl[i,2],])

dist.diff.test[pl[i,1],pl[i,2],]<-distance(test_mu[,pl[i,1],],test_mu[,pl[i,2],]) -

distance(test_mu[,pl[i,1],],null_mu[,pl[i,1],]) - distance(test_mu[,pl[i,2],],null_mu[,pl[i,2],])

}

#summarize posterior

dist.sum<-matrix(NA, nrow=nrow(pl),ncol=7)

probs<-c(0.025,0.5,0.975)

for(i in 1:(ngroups*ngroups)){

dist.sum[i,]<-c(pl[i,1:2],quantile(dist.diff[pl[i,1],pl[i,2],],probs=probs),mode(dist.diff[pl[i,1],pl[i,2],]),mean(dist.diff[pl[i,1],pl[i,2],]))

}

dist.sum<-dist.sum[c(-1,-6,-11,-16),]

colnames(dist.sum)<-c("group","group",paste(probs,"%",sep=""),"mode","mean")

# Calculate probabilities that the distance estimates overlap zero

pdist<-matrix(NA, nrow=ngroups,ncol=ngroups)

for (i in 1:(ngroups*ngroups)){

pdist[pl[i,1],pl[i,2]]<-sum(dist.diff.test[pl[i,1],pl[i,2],]>0)/(nrow(out$sims.list$mu)/2)

}

colnames(pdist)<-groupnames

rownames(pdist)<-groupnames

print(pdist,digits=2)

#######################################################################################################

# Area Overlap

#######################################################################################################

# define objects for mu and sigma values from the posterior

mus<-out$sims.list$mu

sigmas<-out$sims.list$cov

#create a matrix of all pair-wise comparisons

pairs<-pairwise(ngroups)

# Create a loop to 1) calculate the area of over lap for each pair-wise comparison

# and 2) compare that value to each group's respective SEV value

overlap<-matrix(NA, nrow=nrow(mus), ncol=nrow(pairs))

overlap.SEV<-array(1, dim=c(ngroups,ngroups,nrow(mus)))

for(i in 1:nrow(pairs)){

for(j in 1:nrow(mus)){

overlap[j,i]<-CubeCount3D(mus[j,pairs[i,1],],

mus[j,pairs[i,2],],

sigmas[j,,,pairs[i,1]],

sigmas[j,,,pairs[i,2]])

}

overlap.SEV[pairs[i,1],pairs[i,2],]<-overlap[,i]/SEV[,pairs[i,1]]

overlap.SEV[pairs[i,2],pairs[i,1],]<-overlap[,i]/SEV[,pairs[i,2]]

}

#summarize results

overlap.2.5<-matrix(NA, nrow=ngroups, ncol=ngroups)

overlap.50<-matrix(NA, nrow=ngroups, ncol=ngroups)

overlap.975<-matrix(NA, nrow=ngroups, ncol=ngroups)

for (i in 1:nrow(pairs)){

overlap.2.5[pairs[i,1],pairs[i,2]]<-quantile(overlap.SEV[pairs[i,1],pairs[i,2],], probs=0.025)

overlap.50[pairs[i,1],pairs[i,2]]<-quantile(overlap.SEV[pairs[i,1],pairs[i,2],], probs=0.5)

overlap.975[pairs[i,1],pairs[i,2]]<-quantile(overlap.SEV[pairs[i,1],pairs[i,2],], probs=0.75)

}

#####################################################################################################

# Print results

print(SEV.summary,digits=2)

print(p.table, digits=2)

print(mu.p,digits=2)

print(dist.sum,digits=2)

print(pdist,digits=2)

print(overlap.2.5,digits=2)

print(overlap.50,digits=2)

print(overlap.975,digits=2)

**Figure A1.** A. Estimates of **µ** for simulated populations one through four (denoted on right side of panel) for each sample size n=6 to 100. The shaded regions represent the 95% CI for every value of **µ** which were equal within each simulated populations. Black lines indicate the median estimate for each sample size. White lines indicate the true value and are completely obscured by the black lines. B. The power to detect differences in SEV between two populations. Each line shows the power for pairwise comparisons of a population’s SEV with that of population one. We define power as the proportion of model runs in which the probability of the SEV of populations two, three, or four (denoted next to each line) being larger than that of population one was 0.90 or greater.


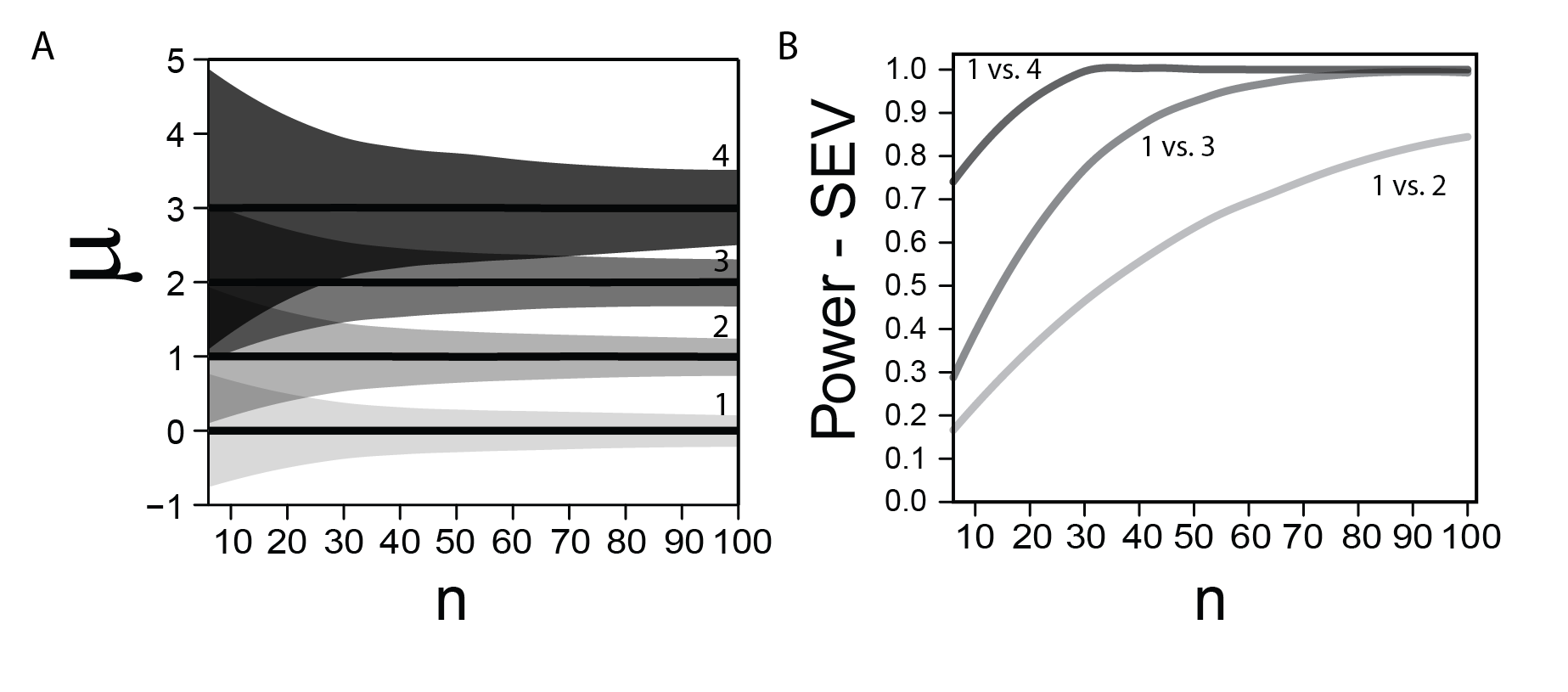

Supplement: Supplementary file 1 — Appendix S1. Additional details on the simulation study. [file ECE3-6-2405-s001.docx]
