# Supplementary material for: Beyond carbon and nitrogen: guidelines for estimating three‐dimensional isotopic niche space
Source: Ecol Evol. 2016 Mar 9;6(8):2405–13. doi: 10.1002/ece3.2013 (PMC4834325; doi:10.1002/ece3.2013)

**Appendix B: Standard ellipsoid model using individual priors on terms within the covariance matrix, Σ**

**Panel B1:** JAGS model code using individual priors on terms of **Σ** (instead of the inverse-Wishart prior presented in the main text). We used a Cholesky decomposition to ensure that **Σ** was positive definite.

# JAGS model

model{

#priors

for (k in 1:ngroups){

mu[k,1] ~ dnorm(0,0.001)

mu[k,2] ~ dnorm(0,0.001)

mu[k,3] ~ dnorm(0,0.001)

# Cholesky decomposition

A[1,2,k] <-0

A[1,3,k] <-0

A[2,3,k] <-0

A[1,1,k] ~ dgamma(0.1,0.1)

A[2,2,k] ~ dgamma(0.1,0.1)

A[3,3,k] ~ dgamma(0.1,0.1)

A[2,1,k] ~ dnorm(0, 0.1)

A[3,1,k] ~ dnorm(0, 0.1)

A[3,2,k] ~ dnorm(0, 0.1)

cov[1:3,1:3,k] <- A[,,k]%*%t(A[,,k])

prec[1:3,1:3,k]<-inverse(cov[,,k])

}

#likelihood

for (k in 1:ngroups){

for(i in 1:maxn){

y[i,1:3,k] ~ dmnorm(mu[k,],prec[,,k])

}#k

}#i

}

**Figure B1:** Standard ellipsoid volumes (SEV) for four populations (corresponding to labels upper right of each panel) with increasing isotopic niche. The black lines represent the median estimated values from 1,000 simulated datasets for each population at each sample size n = 6 through n = 100. White lines represent the true value. Left panels show results using the inverse-Wishart prior (also shown in main text). Right panels show results in which an individual prior is set for each term within the covariance matrix, **Σ**.


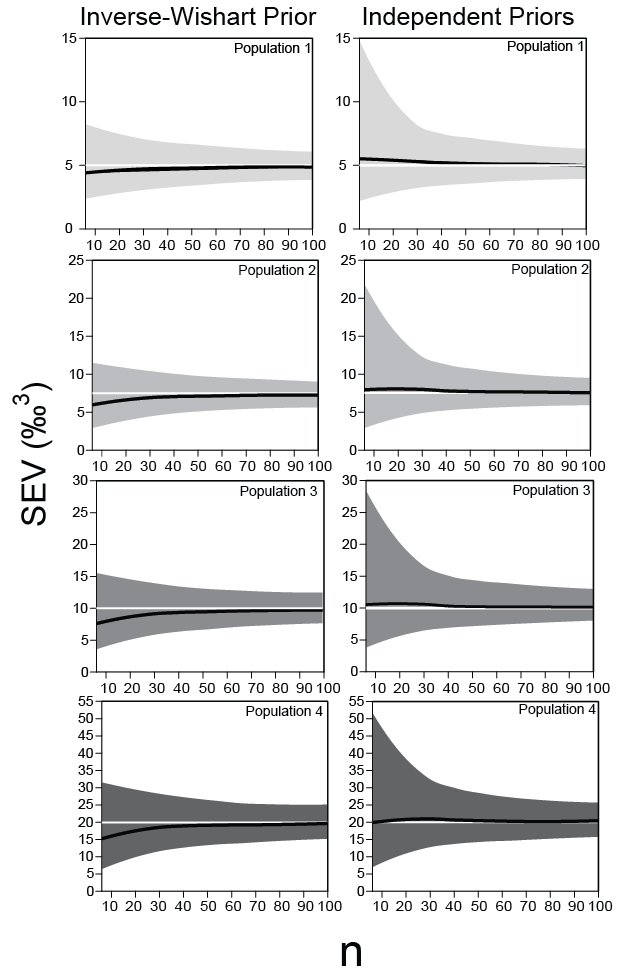


**Figure B2.** Difference between SEV estimates and true values for four populations (corresponding to labels upper right of each panel) with increasing isotopic niche size (SEV for populations 1-4: 5‰^3^, 7.5‰^3^, 10‰^3^, and 20‰^3^ respectively). Black and gray areas represents the 95% CI for the model with individuals priors and the inverse-Wishart prior (respectively) on sigma. Black points indicate means for the individual prior model and white points indicate means for the inverse-Wishart model.


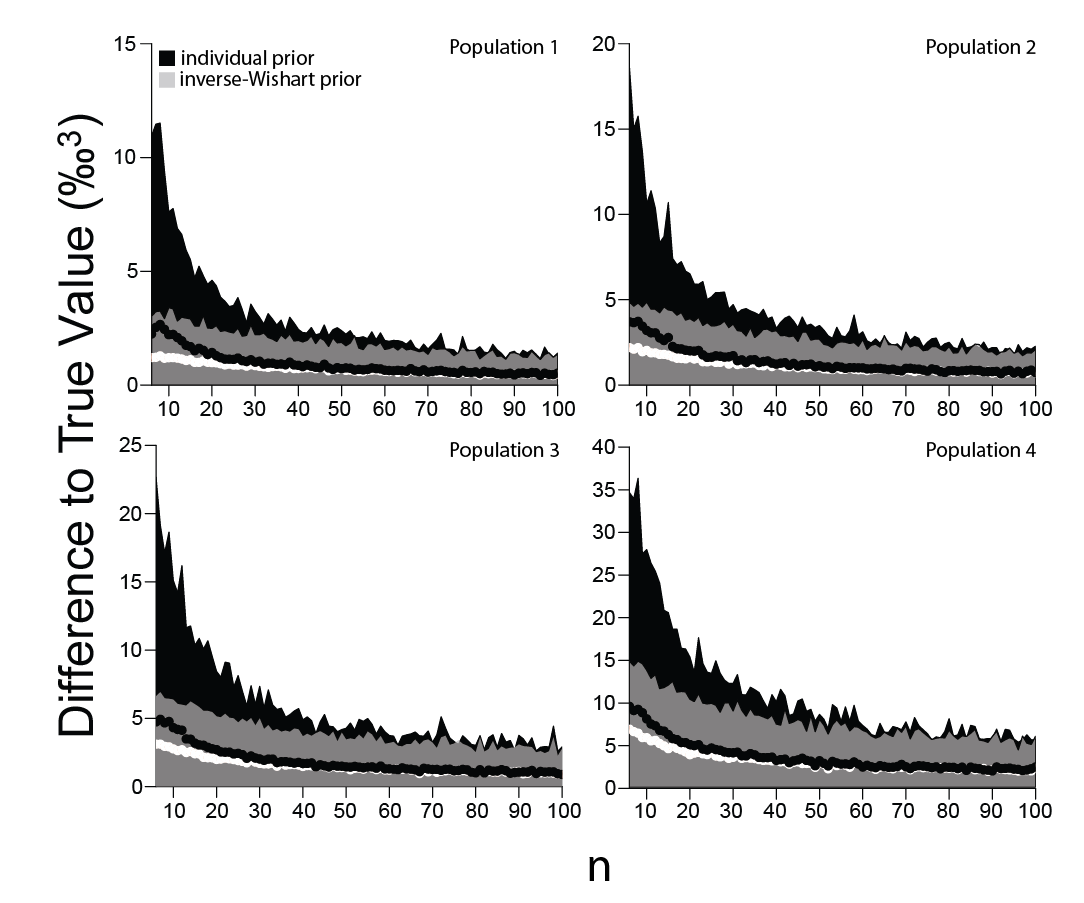

Supplement: Supplementary file 2 — Appendix S2. Standard ellipsoid model using individual priors on terms within the covariance matrix, Σ. [file ECE3-6-2405-s002.docx]
